# Ifosfamide-Induced Metabolic Encephalopathy in 2 Patients With Cutaneous T-Cell Lymphoma Successfully Treated With Methylene Blue

**DOI:** 10.1177/2324709618786769

**Published:** 2018-07-06

**Authors:** Anusha Vakiti, Ravi Pilla, Muhamad Alhaj Moustafa, Jacinth J. Joseph, Aarthi G. Shenoy

**Affiliations:** 1 MedStar Washington Hospital Center, Washington, DC, USA

**Keywords:** ifosfamide-induced encephalopathy, metabolic encephalopathy, methylene blue, neurotoxicity, aprepitant

## Abstract

Ifosfamide, an alkylating agent used in cancer treatments, can cause neurotoxicity. The clinical presentation can range from mild symptoms such as acute confusion to non-convulsive seizures, severe irreversible coma, and death. The benefit of methylene blue use in treating ifosfamide-induced metabolic encephalopathy is not well established. In this article, we present 2 cases of ifosfamide-induced metabolic encephalopathy responsive to methylene blue treatment.

## Introduction

Ifosfamide (a structural analog of cyclophosphamide) is an alkylating agent that is frequently used in the treatment of soft-tissue sarcoma, lymphoma, germ cell tumors, and gynecologic malignancies. There are many well-known side effects of ifosfamide including hemorrhagic cystitis, nephrotoxicity, myelosuppression, and neurotoxicity. Neurotoxicity has been reported in 5-30% of all patients treated with ifosfamide.^1^ The clinical presentation can range from mild symptoms such as confusion to non-convulsive seizures and more overt encephalopathy. Few cases of catastrophic side effects such as severe coma and death have also been reported.^2^ Ifosfamide-induced metabolic encephalopathy (IME) is usually treated by discontinuing the agent, adequate hydration, and in few cases with methylene blue (MB) administration.^3^ The benefit of MB use for treatment of IME is not well established and certainly can be problematic in patients with glucose-6-phosphate dehydrogenase (G6PD) deficiency. Herein, we present two cases of cutaneous T-cell lymphoma in which ifosfamide use induced metabolic encephalopathy and early use of MB was associated with resolution of the symptoms.

## Case 1

A 47-year-old African American male with stage III cutaneous T-cell lymphoma with large cell transformation presented to the hospital with shortness of breath, fatigue, and failure to thrive. The patient had progressed through multiple lines of chemotherapy including EPOCH (etoposide, prednisone, vincristine, cyclophosphamide, and doxorubicin), romidepsin, gemcitabine, brentuximab, and pralatrexate. At inpatient presentation, he had malignant pleural effusions, hypercalcemia, and leukocytosis with eosinophilia. Given the patient’s rapidly worsening clinical course, it was decided to start ifosfamide, carboplatin, and etoposide (ICE) chemotherapy with etoposide 100 mg/m^2^ on days 1 to 3, ifosfamide 5000 mg/ on day 2 infused over 24 hours, and carboplatin with an area under curve (AUC) of 5 on day 3. He was alert and oriented prior to and during the infusion but became delirious on day 3 of the regimen, 6 hours after the completion of the ifosfamide infusion. Physical examination did not reveal any stereotypical movements or twitching. His mental status worsened from initial agitation and confusion to drowsiness and eventually stupor. Diagnostic evaluation for acute mental status change including complete blood count, comprehensive metabolic panel, ammonia level, and computed tomography scan of the head was negative. Infectious workup including blood cultures, urine culture, and chest X-ray did not reveal any infectious process contributing to the altered mental status. Thus, the patient’s acute altered mental status was attributed to IME. He was started on hydration, and 50 mg of intravenous MB was given every 4 hours. His mental status began to improve 16 hours after start of MB and was back to his baseline after 48 hours of treatment (received 12 total doses). The one cycle of ICE chemotherapy temporarily improved his disease but due to neurotoxicity, he was not rechallenged.

## Case 2

A 38-year-old African American female with refractory stage IV mycosis fungoides with large cell transformation was admitted to the hospital for initiation of ICE chemotherapy. Patient had extensive cutaneous and muscle involvement of the lymphoma and had failed multiple lines of chemotherapy regimens including romidepsin and rituximab, brentuximab, and gemcitabine. Laboratory testing prior to initiation of ICE therapy was normal except for low serum albumin level of 1.7 g/dL. Patient received ICE therapy with etoposide 100 mg/m^2^ on days 1 to 3, ifosfamide 5000 mg/m^2^ on day 2 infused over 24 hours, and carboplatin with an AUC of 5 on day 3. She was premedicated with aprepitant for prevention of nausea. She was alert and oriented prior to and during the infusion but became lethargic, somnolent, and confused within 6 to 8 hours of completion of ifosfamide infusion. Physical examination was significant for random jerky movements of both upper and lower extremities, twitching of the right eye, and somnolence. The patient’s evaluation for sudden neurological changes included complete blood count, comprehensive metabolic panel, computed tomography scan of the head, and infectious workup (blood cultures, urine culture, and chest X-ray), all of which were negative. She received naloxone with no reversal of mental status changes. Based on the timing of the infusion and change in mental status, patient was diagnosed with IME and MB was immediately initiated at a dose of 50 mg every 4 hours. An improvement in the patient’s mental status was noticed within 12 hours of initiation of MB, and the patient was back to her baseline in 72 hours (received 18 total doses). Given the neurotoxicity, patient was not rechallenged with ifosfamide and was switched to a different chemotherapeutic regimen.

## Discussion

Ifosfamide is a prodrug which undergoes hepatic activation by cytochrome P450 (CYP450) to its cytotoxic metabolites. The neurotoxicity of ifosfamide is dose dependent and is caused by its metabolites, mainly chloroacetaldehyde (CAA).^4^ CAA can cross the blood-brain barrier and cause neurotoxic effects by inhibition of mitochondrial oxidative phosphorylation and depletion of glutathione from the central nervous system.^4^ Multiple studies have evaluated factors that increase the risk for ifosfamide toxicity and have concluded that female gender, low serum albumin, hepatic or renal dysfunction, previous cisplatin use, and interactions with drugs which increase ifosfamide metabolism in the liver (such as phenobarbital) increase the risk of toxicity.^5-7^ Concomitant use of aprepitant has also been shown to increase the risk of IME.^8-10^ In a retrospective study done by Howell et al, 75% of the total patients who developed ifosfamide-associated neurotoxicity also received aprepitant.^9^ In case 2, the patient received aprepitant, which we believe contributed to IME along with other risk factors such as female gender and low serum albumin level. Both the patients in our case report were African Americans and their genotype could be contributing to the variability in hepatic CYP450 oxidase mediated drug metabolism, resulting in susceptibility to toxicity at therapeutic doses.

IME has a wide clinical spectrum as outlined in the national cancer institute (NCI) toxicity grading for encephalopathy. Approximately 40-50% of the patients fall into grade I (mild) or grade II (moderate) toxicity, 40% of the patients into grade III (severe) toxicity, and 10-20% of the patients into grade IV (coma) toxicity.^11^ The patients in our case report experienced grade III (severe) neurotoxicity, per NCI toxicity grading. Electroencephalography was not performed in either case as the IME was uncomplicated by non-convulsive seizures and began improving within 12 to 16 hours without the introduction of an anticonvulsant. However, non-convulsive seizures should be considered in severe cases and where no improvement is seen over time even after cessation of the ifosfamide infusion. Few studies have reported extrapyramidal symptoms and presentation of myoclonus and opisthotonic posturing.^12^ There are case reports of persistent organic brain syndrome lasting 10 weeks posttreatment and a report of irreversible encephalopathy,^13^ raising concern for potentially longer natural course of untreated toxicity.^14^ Irreversible neurological damage in the form of cerebral atrophy and cerebellar degeneration have also been reported.^15,16^ In few cases, IME can be fatal and in patients with grade III or IV toxicity can lead to death.^2^

The mainstay of management in patients with suspected IME is by immediate cessation of the drug administration and adequate hydration. The symptoms of IME usually resolve within 48 to 72 hours after discontinuing the drug in patients with grade I or II toxicities. However, patients with higher grades of toxicity may exhibit symptoms for longer duration and in few cases may have irreversible neurological damage. Patients with severe symptoms may benefit from the use of MB. The use of MB in ifosfamide neurotoxicity treatment was first reported in 1994. It acts as an electron acceptor and the suggested mechanism of action of MB is that it inhibits the extrahepatic monoamine oxidation of chloroethylamine to CAA, thus preventing the formation of CAA. Several cases have reported the successful resolution of neurological symptoms following the use of MB.^17-20^ In a study by Pelgrims et al, of the 8 patients who received MB for IME, 4 recovered fully within 24 hours, which is shorter than the historic 48 to 72 hours, thus suggesting that MB likely has a role in reducing the duration of symptoms.^3^ Although its use is becoming more prevalent, there is no strong evidence for its efficacy as clinical trials have not been conducted. The rarity of the problem makes it difficult to study it in a controlled trial setting.

The prompt reversal of altered mental status after 12 to 18 doses of MB and return to baseline in 48 to 72 hours, despite severe toxicity in our patients, makes us suspect that MB had a role in the resolution of the IME. Although toxicities are reversible, given the potential for irreversible neurological damage and mortality rate of 10-30% in untreated IME, we would recommend the use of MB, particularly in patients with NCI grade III or IV encephalopathy. Prophylactic use of MB with ifosfamide has been explored in patients with history of neurologic toxicity who need to be rechallenged with ifosfamide. In these cases, patients who needed to be rechallenged with ifosfamide received MB prophylactically without any return of symptoms of neurotoxicity.^19-21^

Common side effects of MB include blue-green discoloration of urine, nausea, and bladder irritation. Although rare, more severe side effects include syncope, paresthesia, and cardiac arrhythmias. Few cases of serotonin syndrome have been reported with concomitant use of MB with selective serotonin reuptake inhibitors, serotonin and norepinephrine reuptake inhibitors, and monoamine oxidase inhibitors.^22,23^ It can also cause hemolytic anemia characterized by Heinz body formation, especially in patients with G6PD deficiency. Its use is contraindicated in patients with G6PD deficiency and in pregnant women.

In conclusion, the successful treatment of severe IME with MB in our patients contributes further to the existing reports for its benefit in this setting. Given its low cost, benign side effect profile, and wide availability, we suggest that use of MB be considered when IME is suspected. Further studies comparing patients with untreated IME to patients who were treated with MB are needed to predict long-term outcomes and establish its efficacy.
